# Improved Screening of Monoclonal Gammopathy Patients by MALDI-TOF Mass Spectrometry

**DOI:** 10.1021/jasms.3c00166

**Published:** 2023-11-23

**Authors:** Lukáš Pečinka, Monika Vlachová, Lukáš Moráň, Jana Gregorová, Volodymyr Porokh, Petra Kovačovicová, Martina Almáši, Luděk Pour, Martin Štork, Josef Havel, Sabina Ševčíková, Petr Vaňhara

**Affiliations:** † Department of Chemistry, Faculty of Science, Masaryk University, Kamenice 5, 625 00 Brno, Czech Republic; ‡ International Clinical Research Center, St. Anne’s University Hospital Brno, Pekařská 53, 656 91 Brno, Czech Republic; § Babak Myeloma Group, Department of Pathophysiology, Faculty of Medicine, Masaryk University, Kamenice 3, 625 00 Brno, Czech Republic; ∥ Department of Histology and Embryology, Faculty of Medicine, Masaryk University, Kamenice 3, 625 00 Brno, Czech Republic; ⊥ Research Centre for Applied Molecular Oncology (RECAMO), Masaryk Memorial Cancer Institute, Žlutý kopec 7, 602 00 Brno, Czech Republic; # Department of Clinical Hematology, University Hospital Brno, Jihlavská 20, 625 00 Brno, Czech Republic; ¶ Department of Internal Medicine, Hematology and Oncology, University Hospital Brno, Jihlavská 20, 625 00 Brno, Czech Republic

**Keywords:** MALDI-TOF mass spectrometry, multiple myeloma, plasma cell leukemia, monoclonal gammopathy, principal component analysis, machine learning, partial least-squares-discriminant analysis, molecular profiling, fingerprinting

## Abstract

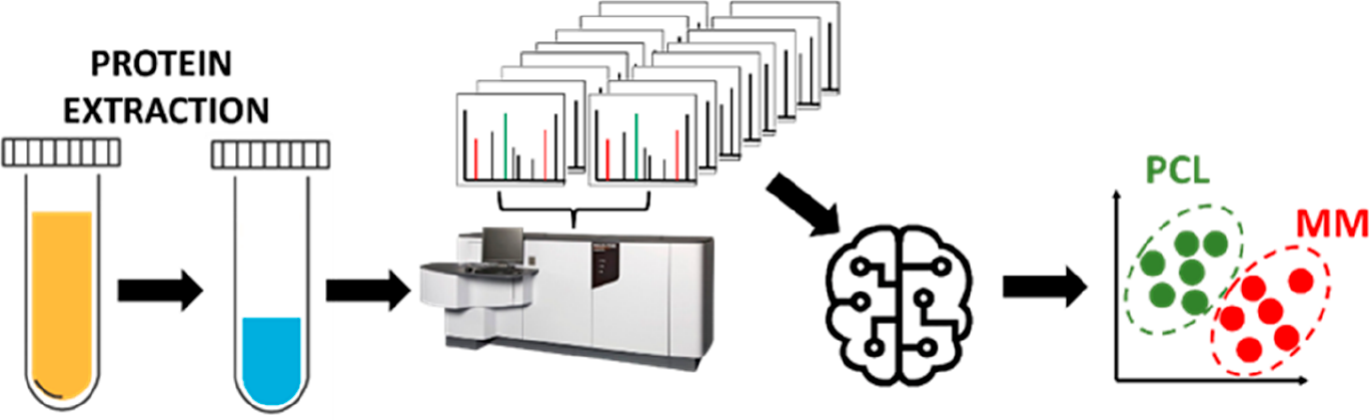

Monoclonal gammopathies are a group of blood diseases characterized by presence of abnormal immunoglobulins in peripheral blood and/or urine of patients. Multiple myeloma and plasma cell leukemia are monoclonal gammopathies with unclear etiology, caused by malignant transformation of bone marrow plasma cells. Mass spectrometry with matrix-assisted laser desorption/ionization and time-of-flight detection is commonly used for investigation of the peptidome and small proteome of blood plasma with high accuracy, robustness, and cost-effectivity. In addition, mass spectrometry coupled with advanced statistics can be used for molecular profiling, classification, and diagnosis of liquid biopsies and tissue specimens in various malignancies. Despite the fact there have been fully optimized protocols for mass spectrometry of normal blood plasma available for decades, in monoclonal gammopathy patients, the massive alterations of biophysical and biochemical parameters of peripheral blood plasma often limit the mass spectrometry measurements. In this paper, we present a new two-step extraction protocol and demonstrated the enhanced resolution and intensity (>50×) of mass spectra obtained from extracts of peripheral blood plasma from monoclonal gammopathy patients. When coupled with advanced statistics and machine learning, the mass spectra profiles enabled the direct identification, classification, and discrimination of multiple myeloma and plasma cell leukemia patients with high accuracy and precision. A model based on PLS-DA achieved the best performance with 71.5% accuracy (95% confidence interval, CI = 57.1–83.3%) when the 10× repeated 5-fold CV was performed. In summary, the two-step extraction protocol improved the analysis of monoclonal gammopathy peripheral blood plasma samples by mass spectrometry and provided a tool for addressing the complex molecular etiology of monoclonal gammopathies.

## 1 Introduction

Mass spectrometry (MS) with matrix-assisted laser desorption/ionization and time-of-flight detection (MALDI-TOF MS) is a sensitive and frequently used technique in various experimental and clinical research fields for detection of molecular entities ranging from small molecules and metabolites to peptides, proteins, and lipids, even in subfemtomole amounts.^1^ MALDI-TOF MS is applicable to virtually all types of biological samples including cell extracts, body fluids, and tissue samples.^2,3^ In liquid biopsies of peripheral blood, MALDI-TOF MS provides an attractive diagnostic and research tool. However, the type of sample and its preparation determine the reproducibility and credibility of MALDI-TOF MS analysis. This is particularly true for blood dyscrasias that are linked to changes in biophysical parameters of blood, such as viscosity and concentration levels of various molecules (ions, small organic molecules, proteins, and lipids) that can interfere with precise MS measurements.^4^

Technical approaches and methods for studying the plasma peptidome, small proteome, and fragmentome of large proteins by MALDI-TOF MS were excellently reviewed by Hortin.^5^ When compared to liquid-chromatography MS (LC-MS), MALDI-TOF MS analysis of peripheral blood plasma does not require laborious and time-consuming sample preparation steps. However, the analysis of peripheral blood plasma often suffers from high salinity of the sample and subsequent ion suppression effects. The abundant plasma proteins, such as albumin, strongly suppress the signal of proteins at low concentration levels.^3^ Extraction and hydrolysis techniques were thus developed to reduce ion suppression effects and improve MS analysis. Various extraction methods involving one or two steps were developed based on the different focus of the study and the sample type.^5−8^ Acetonitrile added to plasma samples efficiently precipitates large abundant proteins, such as albumin; however, smaller proteins and peptides stay in solution and can be analyzed by MS.^9,10^ Lin et al. used acetonitrile for protein extraction followed up by acid hydrolysis of albumin using trifluoroacetic acid for characterization of potential protein biomarkers in peripheral blood plasma of patients suffering from major depressive disorder.^3^ Chertov et al.^10^ used two volumes of acetonitrile added to mouse serum samples for significant improvement of mass spectra and for subsequent detection of two protein markers in the blood serum extract from the tumor-bearing mice. In monoclonal gammopathy patients, the abnormal biochemical composition of blood plasma decreases the quality of the MS measurements and compromises further analyses.

Monoclonal gammopathies are a group of diseases characterized by large quantities of abnormal immunoglobulins in peripheral blood and urine of patients. Multiple myeloma (MM) is the second most common hematological malignancy of the elderly; the median age of diagnosis is 68 in men and 70 in women in the Czech Republic.^11^ It is a heterogeneous disease characterized by infiltration of the bone marrow by malignant clonal plasma cells, suppression of physiological hematopoiesis and bone lesions, and production of monoclonal immunoglobulin. Plasma cell leukemia (PCL) is a rare, aggressive disease with a poor prognosis. PCL is characterized by circulation of malignant plasma cells in peripheral blood.^12−14^ Although both MM and PCL involve the same malignant cell type, PCL differs from MM in some clinical and laboratory parameters such as lower incidence of bone lesions or higher incidence of splenomegaly or hepatomegaly. Whether the etiopathology of PCL and MM is the same, or different mechanisms are involved in the development of MM and PCL, remains unclear.^15^ Spectral profiling by MALDI-TOF MS can reveal informative molecular patterns and contribute to a better understanding of the biological background as well as diagnostics or follow-up of PCL and MM patients. Recently, several studies have implemented MALDI MS to monitor various biomarkers in monoclonal gammopathies.^16−21^ In particular, abnormal immunoglobulin (M-protein) is produced in large quantities by malignant plasma cells in both MM and PCL patients. Detection and isotyping of the paraprotein by electrophoresis are common in clinical practice, but alternative methods including mass spectrometry have been successfully evaluated.^17−19^ Eveillard et al. compared the MALDI MS assay to the panel of routine methods (serum protein electrophoresis, immunofixation, and serum-free light chain testing) in newly diagnosed MM patients treated with daratumumab-based combination therapy.^21^ These results show an improvement in the detection rate of all isotypes of M proteins in MM patients when MALDI-TOF MS is used. In addition, Barceló et al. have used the MALDI MS fingerprint of small proteome 2–10 kDa coupled with machine learning to predict the presence of monoclonal gammopathy with nearly 90% accuracy, sensitivity, and specificity.^16^ This suggests that spectral fingerprints can be used for diagnostics and discrimination of various monoclonal gammopathies using liquid biopsies.

We have previously shown that spectral fingerprinting of peripheral blood plasma by MALDI-TOF MS coupled with machine learning can reveal informative molecular patterns and clearly discriminate the healthy donors (HD) from MM patients.^22^ Therefore, we wondered if the same approach can be used to distinguish two monoclonal gammopathies, MM and PCL samples, just by differences in spectral patterns. To the best of our knowledge, no publications describing spectral differences between MM and PCL using liquid biopsies from peripheral blood have been published, and only a limited number of publications have addressed the profiling and classification of HD and MM patients.^17,22^

In this study, proteins in peripheral blood plasma samples were precipitated using organic solvent, acetonitrile (ACN), in the first extraction step and resuspended in ACN:H_2_O supplemented with trifluoroacetic acid (TFA) in the second extraction step. Protein extracts were analyzed using MALDI-TOF MS to distinguish MM patients from PCL patients. The MS results were further analyzed using the multivariate statistical methods: principal component analysis (PCA), partial least-squares-discriminant analysis (PLS-DA), and orthogonal PLS-DA (OPLS-DA). Machine learning (ML) based on decision tree (DT), random forest (RF), k-nearest neighbors (k-NN), partial least-squares discriminant analysis (PLS-DA), and artificial neural network (ANN) algorithms were designed to correctly classify MM and PCL patients.

## 2 Methods

### 2.1 Materials and Instruments

Trifluoroacetic acid, sinapic acid (SA), α-cyano-4-hydroxycinnamic acid, and 9-aminoacridine were purchased from Sigma-Aldrich (Steinheim, Germany). Acetonitrile was purchased from Penta (Prague, Czech Republic). Peptide calibration mixture ProMix1 was purchased from LaserBio Laboratories (Valbonne, France).

Benchtop centrifuge Eppendorf Minispin Plus (Eppendorf, Germany) and an ultrasonic bath Laboratory 3 (Thermo Fisher Scientific, USA) were used.

### 2.2 Sample Collection

Samples of peripheral blood plasma from 15 healthy donors (HD), 20 multiple myeloma (MM), and 13 plasma cell leukemia (PCL) patients were included in the study. MM and PCL samples were obtained at the time of diagnosis. HD, MM, and PCL samples were obtained from University Hospital Brno. All patients signed informed consent forms approved by the ethics committee of the hospital following the Declaration of Helsinki. All plasma samples were handled as previously described and stored at −80 °C.^23^ The extracts of the peripheral blood plasma were stored at −20 °C.

### 2.3 Protein Extraction

Plasma samples were thawed on ice and then centrifuged (5 min at 14 500 rpm) to remove any cellular detritus. In the first extraction step, 50 μL of ACN was added to the 25 μL of the plasma sample.^3^ The mixture of plasma and ACN was then sonicated in an ultrasonic bath for 10 min, followed by centrifugation for 5 min at 14 500 rpm. The collected supernatant was discarded, and the precipitate was retained and used in the next step. Then, the second extraction step was performed by adding 50 μL of 50% ACN supplemented with 0.1% TFA to the sample, followed by sonication and centrifugation. The collected supernatant was analyzed by MALDI-TOF MS.^22,24^ As a control, samples of original plasma were diluted 10 times by using double distilled water to provide a direct comparison with the two-step protein extraction protocol.

### 2.4 Acquisition of Mass Spectra

The collected extract was used for MALDI-TOF MS measurements when mixed in a 1:1 ratio with the matrix, which yielded the best results, i.e., 20 mg/μL SA dissolved in 50% ACN with 2.5% TFA. Then, five technical replicates of each sample (2 μL) were spotted on a MALDI metal target plate as described previously.^22,24^ Different MALDI matrices, different variations of the matrix (varying concentrations of SA and ratios of components in the solvent), spotting volumes, and different measurement conditions were tested (data available upon request). After drying at room temperature, the target plate was transferred into a MALDI-7090 TOF (Shimadzu, Japan) mass spectrometer equipped with a 2 kHz ultrafast solid-state UV laser (Nd:YAG: 355 nm), and variable beam focuses from 10 μm to >100 μm. Mass spectra were recorded in the linear positive ion mode, in the mass region of 2–20 kDa, pulse extraction was set to 12.5 kDa, frequency of the laser was 1 kHz, and the laser diameter was 100 μm. In total, 5 profiles from 1000 points were accumulated to record 1 mass spectrum. Calibration was performed externally using the protein calibration mix 1 (ProMix1) 2.8–17 kDa ions.

### 2.5 Processing of Mass Spectra and Multivariate Statistical Analysis

Raw mass spectra in the mzml format were preprocessed using R (4.0.4) to detect differentially expressed species among mass spectra. MALDIquant package, MALDIrppa, and subsequently analysis using several R packages enabling multivariate statistical modeling were used as described in Vaňhara et al.^24^ Before spectra preprocessing, low-quality spectra were identified using semiautomatic screening implemented in the MALDIrppa package. The procedure is based on robust scale estimators of median intensities and derivative spectra.^25^

The spectral preprocessing workflow followed standard procedures adopted from the MALDIquant package: quality control, transformation, and smoothing (Savitzky-Golay filter) with halfwindowSize function = 100, baseline correction (statistics-sensitive nonlinear iterative peak-clipping, SNIP) with 500 iterations, intensity calibration (∑*X*_*i*_ = 1, where *X_i_* represents intensities of corresponding peaks in mass spectra), spectra alignment (removing the nonsystematic shift in technical replication acquired on a different day), trimming (2–20 kDa), and peak detection using a MAD noise estimation algorithm with signal-to-noise = 10 and a half-window size = 20.^26−28^ The feature matrix of detected peaks was constructed only from the peaks that were detected in at least 10% of total mass spectra. The limit of 10% was set to avoid artifacts in mass spectra, which can affect further analysis and decrease the accuracy of classification. Peak lists for all mass spectra were converted to the feature matrix. The established matrix *m* × *n* consists of spectral data, where *m* represents selected *m*/*z* values and *n* is the IDs of individual samples. The *i*-th row of the matrix (*n*) shows the intensities of selected peaks (*m*) of the *i*-th samples (mixture). The feature matrix reduces the data from the original *n* × 400 000 to the *n* × 165. An established matrix of spectral data was used for further multivariate statistical methods and the development of selected classifiers.

Unsupervised (PCA) and supervised (PLS-DA, RF, DT, and ANN) ML algorithms were performed in an R environment using the following R libraries. Namely, factoextra (PCA), corrplot, and stats (hierarchical clustering) and rpart, mdatools, and caret packages were used to construct the unsupervised and supervised ML algorithms, respectively.

## 3 Results and Discussion

### 3.1 Optimization of Protein Extraction of Plasma Samples

We aimed to reduce the unwanted interference of highly abundant plasma proteins and high salinity with MS analysis of the monoclonal gammopathy samples. We introduced a two-step protocol, where the lipids and other low-molecular compounds were removed in the first step and the high-mass protein, keratins, and the residual cellular detritus in the second step, leading to a homogeneous sample suitable for further analysis.

Different extraction protocols were investigated: one-step extraction using methanol:chloroform (1:2) and 0.2 volume equivalents of water, the Folch method (i.e., methanol, chloroform, and water in a ratio of 8:4:3), precipitation using different concentrations of ACN (50–100%) followed by resuspension of the precipitate in TFA of varying concentration (0.1–25%), or a solution of tris(2-carboxyethyl)phosphine and 11% formic acid, followed by a two-step extraction with ACN and subsequent extraction with ACN containing varying concentrations of TFA.^5,6,8^ However, these protocols did not bring satisfactory outputs in monoclonal gammopathy samples. Even when these protocols were further modified, e.g., by pooling of supernatant after each extraction step, preconcentration of extracts, sample evaporation using a vacuum concentrator, or the use of ZipTip desalting pipetting tips, there was a minimal improvement of MS signal. Moreover, these modifications are consistently time-consuming or significantly more expensive compared to organic extraction.

Finally, in the first extraction step, the ratio of ACN to peripheral blood plasma 2:1 (specifically 50 μL of 100% ACN to 25 μL of the plasma sample) was found to be the most effective.

In the second step, 50% ACN was found to be optimal; different percentages of TFA in the 50% ACN were evaluated (Figure S1). TFA concentration was tested in the range of 0–5%. With increasing concentration of TFA, the hydrolysis of proteins was potentiated; therefore, the mass spectra fingerprints changed, the overall intensity of mass spectra decreased, and the number of detected signals was significantly lower (data not shown). Also replacing 50% ACN with water led to significantly worse results (Figure S2). Finally, combination of 50% ACN with 0.1% TFA in the second extraction step shows optimal elution capacity for proteins that are detected in mass spectra. The two-step extraction protocol is schematically shown in Figure S3.

**Figure 1:**
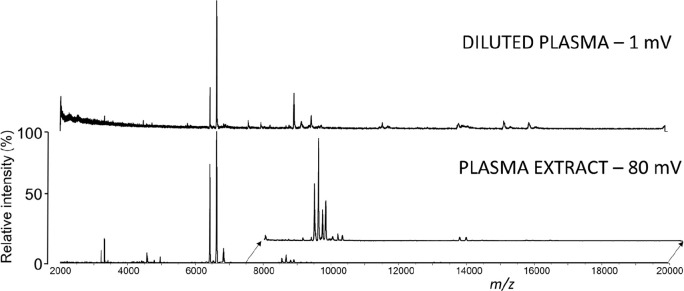
Representative mass spectra of the PCL plasma sample before and after extraction in the *m*/*z* range 2–20 kDa.

Using the same PCL patient sample, the mass spectra of the control, double distilled water-diluted plasma, and the plasma extracts were directly compared. Intensity across the whole *m*/*z* range increased approximately 50 times when the two-step extraction protocol was used (Figure 1). The background noise decreased, and the number of detected *m*/*z* values increased approximately 2 times compared to the diluted plasma. No *m*/*z* values were missing after extraction, indicating that only large proteins (e. g., albumin, keratin) together with remaining impurities and salts were removed.

### 3.2 Analysis of Robustness and Repeatability

#### Robustness in Repeated Freeze–Thaw Cycles

Some biological compounds (peptides and proteins) are more sensitive to repeated freeze–thaw cycles than others, which may influence results when the extract is reused after freezing. Three randomly selected samples from each diagnosis and three control samples were obtained after the two-step extraction protocol. Mass spectra were recorded from freshly prepared aliquots of the blood plasma of three different PCL patients. Then, the same samples were frozen at −20 °C, thawed at room temperature, processed for MS analysis, and frozen again at −20 °C. These cycles were repeated five times; after each cycle, mass spectra were acquired. No significant changes in mass spectral fingerprints were observed, and only negligible oscillation of intensities around the median value was present.

#### Repeatability

**Figure 2:**
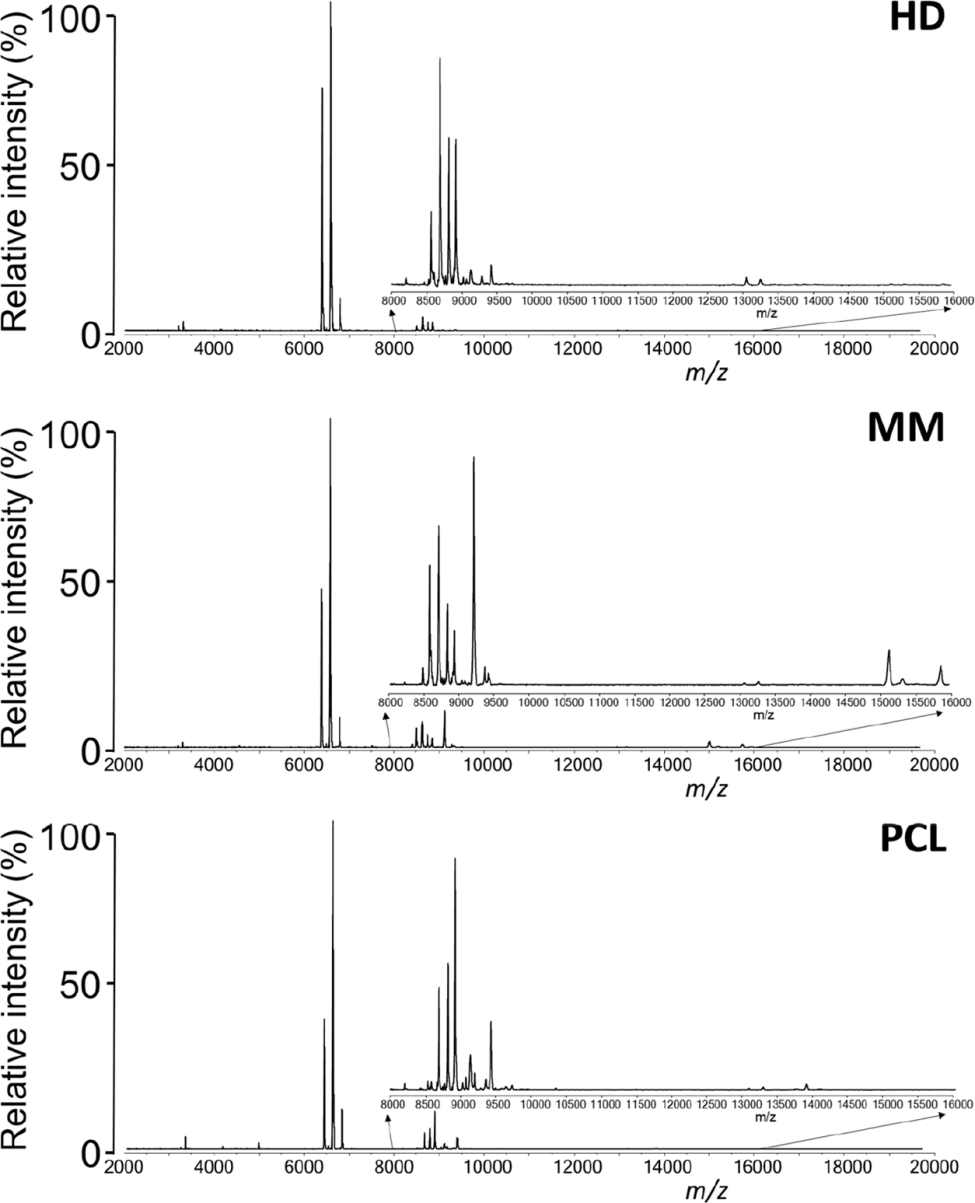
Representative mass spectra of HD, MM, and PCL plasma extracts. The inset shows details of mass spectra fingerprints (8–16 kDa).

The repeatability of the two-step extraction protocol was investigated using the independently frozen aliquots of peripheral blood plasma of a single PCL patient. Extraction and recording of mass spectra were also performed independently on different days using different batches of solvents and matrices. As a result, mass spectra did not differ significantly, confirming the high robustness of the method (Figure S4). Then, we compared the mass spectra of HD, MM, and PCL plasma extracts processed by the two-step extraction protocol and identified differences in mass spectra specific for HD, MM, and PCL (Figure 2).

### 3.3 Multivariate Statistics and Machine Learning Algorithms

**Figure 3:**
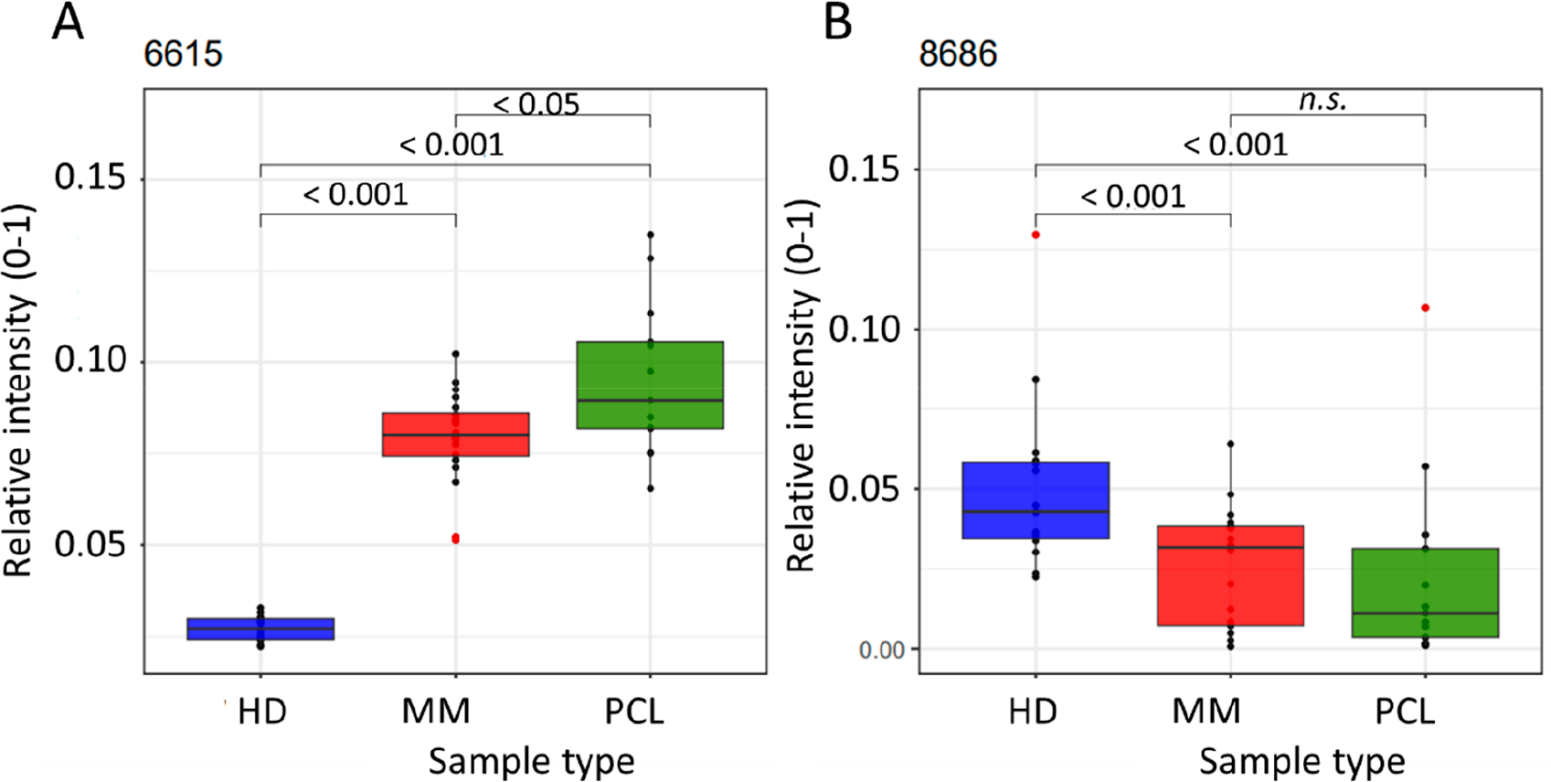
Box plots for selected *m*/*z* values, 6615 Da (A) and 8686 Da (B). The median is indicated by a line. Blue, red, and green box plots indicate healthy donors (HD), multiple myeloma patients (MM), and plasma cell leukemia patients (PCL), respectively.

The principal component analysis (PCA) compared all signals (*n* = 165) which met the conditions that were specified above. The *m*/*z* values that were different in the HD, MM, and PCL groups were identified. For illustration, two molecular entities at *m*/*z* 6615 and 8686 that contributed significantly to the explained variability of PCA are shown in Figure 3.

**Figure 4:**
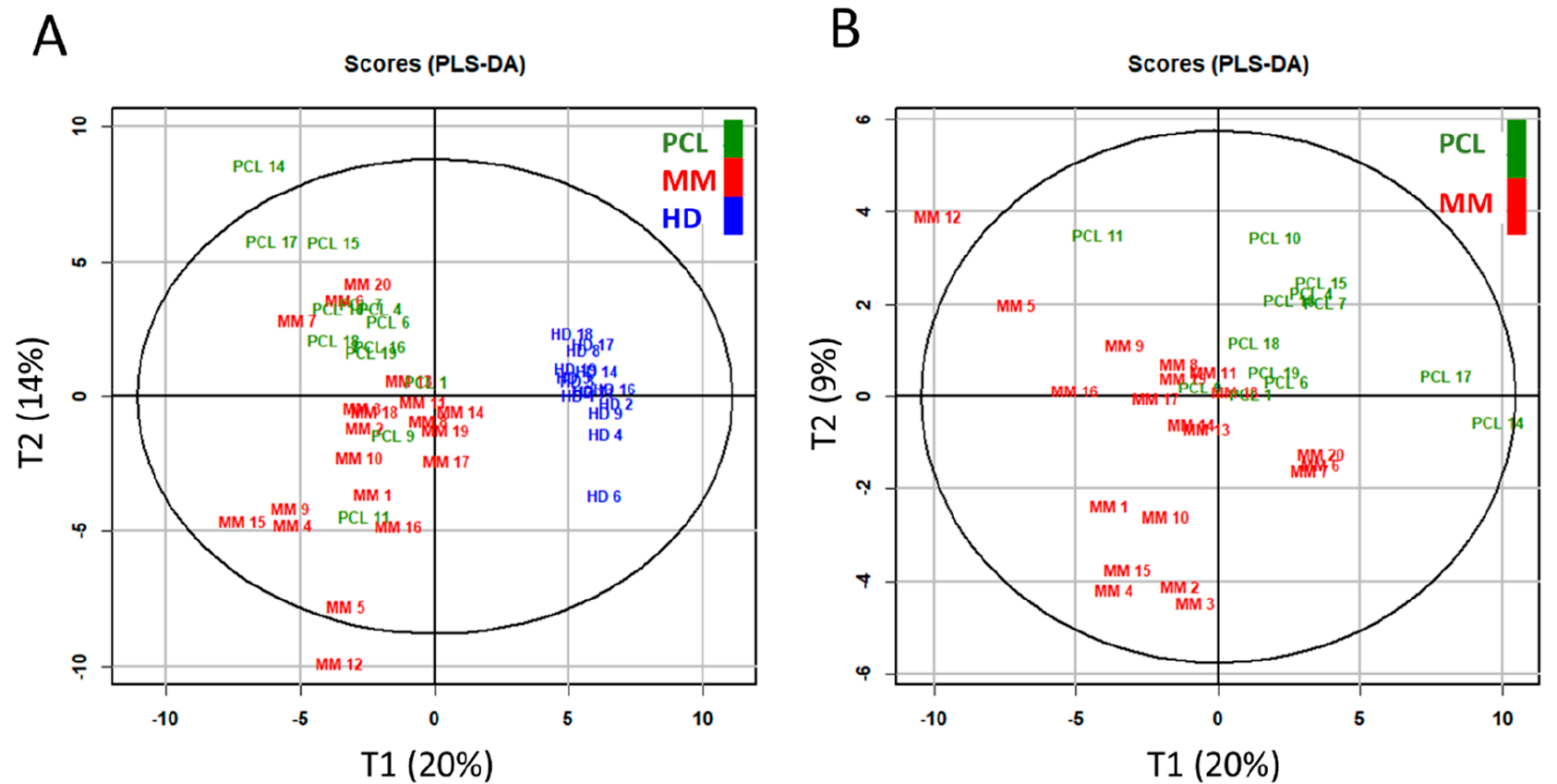
PLS-DA score plot of analysis from plasma samples. Blue, red, and green marks indicate the healthy donors (HD), multiple myeloma patients (MM), and plasma cell leukemia patients (PCL), respectively. PLS-DA provided three groups (A) and two groups (B).

To prove that mass spectra of HD indeed differ from MM and PCL samples, data sets containing all signals were analyzed. Distinct clusters between HD and MM/PCL patients were revealed by using both the unsupervised PCA analysis and supervised PLS-DA (Figure 4A). However, clear separation between MM and PCL was not achieved when the HD samples were included to the analysis. The difference between HD and all patients samples was much bigger than between MM and PCL patient samples, and the variability between MM and PCL groups was suppressed. Therefore, MM and PCL data sets were analyzed separately from HD; MM and PCL clusters were visualized in Figure 4B. In this reduced analysis, the MM and PCL were discriminated better.

**Figure 5:**
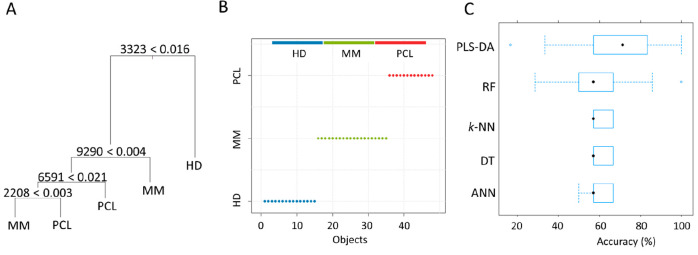
Structure of the DT classification model (A). Accuracy for the training set (B). Comparison of established classifiers based on the accuracy (C).

To verify whether the classification of the selected sample groups (HD, MM, and PCL) can be predicted solely on the basis of computationally processed data, ML algorithms were applied to the data. Due to the size of the patients’ cohorts, cross-validation (CV) of the data set was performed. Leave-one-out cross-validation with 10 times repetition was used to train five different ML classification algorithms: PLS-DA, k-NN, DT, RF, and ANN. These algorithms are particularly suitable for analysis of small data sets and do not require any specific assumptions. The trained models were then evaluated based on the overall accuracy of the prediction. An example of a DT model structure containing four nodes with four dominant variables, where the variable labels correspond to the *m*/*z* values is shown in Figure 5A. For illustration, variable 3324 discriminates well the HD samples, whereas variables 2208, 6591, and 9290 perform well in discriminating MM and PCL samples. Predictions by RF and the PLS-DA achieved the best performance on the full training data set with 96.3% (CI = 97.3–99.6%) and 100% (CI = 93.4–100%) accuracy, respectively (Figure 5B). The optimized structures contained 20 components in PLS-DA and 10 000 trees and a combination of a maximum 10 variables in RF. The accuracy of all models based using CV is summarized in Figure 5C. The best overall accuracy reached the model using PLS-DA was 71.5% accuracy (95% confidence interval, CI = 57.1–83.3%).

## 4 Conclusions

In this work we developed a two-step extraction protocol for improved MALDI-TOF MS analysis of peripheral blood plasma of the two closely related monoclonal gammopathies, MM and PCL. While the MALDI-TOF MS measurement of diluted samples of peripheral blood plasma clearly discriminates between the healthy donors and monoclonal gammopathy patients, it fails in the case of MM and PCL. The published protocols for protein extraction from normal peripheral blood plasma show generally a high efficacy; however, in MM and PCL plasma samples, standard protocols are not efficient, presumably due to different chemical compositions and biophysical properties of monoclonal gammopathy plasma. Here, we achieved a 50-fold increase in intensities of mass spectra by using a two-step extraction protocol requiring less than 25 μL of plasma using 100% ACN in the first step and 50% ACN with 0.1% TFA in the second step. The repeatability of the two-step extraction protocol remains high even after cycles of repeated thawing and freezing of the sample extracts. The statistical evaluation by PCA, PLS-DA, and ML algorithms demonstrated that our proposed procedure could discriminate samples originating from HD, MM, and PCL patients with a high accuracy. A statistical model based on PLS-DA achieved the best performance with 71.5% accuracy (95% confidence interval, CI = 57.1–83.3%) when the 10× repeated 5-fold CV was performed. In summary, we demonstrated for the first time that our method provides high-quality mass spectra that provide unbiased discrimination of two closely related monoclonal gammopathies, reveal them as two different entities based on spectral fingerprinting, and can therefore contribute to the detailed characterization and etiology of MM and PCL.

## Data Availability

The data sets generated during and/or analyzed during the current study are available from the corresponding author upon request.

## Supporting Information Available

Figure S1: Effect of varying concentration of TFA in the second extraction step on quality of mass spectra of peripheral blood plasma PCL patients; Figure S2: Effect of 50% ACN vs 0% ACN in the second extraction step on quality of mass spectra of PCL peripheral blood plasma; Figure S3: Graphical overview of protein extraction; Figure S4: Documenting the reproducibility of the two-step extraction protocol by visualization of mass spectra recorded from four different aliquots of peripheral blood plasma extracts from a single PCL patient (PDF)

## Notes

Ethics Statement: This study was conducted in accordance with the current version of the Helsinki Declaration. This research has been approved by the Ethics committee of the University Hospital Brno. Informed consent was obtained from all subjects involved in the study.
