# Supplementary material for: Improved Screening of Monoclonal Gammopathy Patients by MALDI-TOF Mass Spectrometry
Source: J Am Soc Mass Spectrom. 2023 Nov 23;34(12):2646–53. doi: 10.1021/jasms.3c00166 (PMC10704583; doi:10.1021/jasms.3c00166)
Supplement: Supplementary file 1 — js3c00166_si_001.pdf [file js3c00166_si_001.pdf]

Supporting Information

Improved screening of monoclonal gammopathy patients by MALDI TOF mass spectrometry

Lukáš Pečinka[a,b,] , Monika Vlachová[c], Lukáš Moráň[d,e], Jana Gregorová[c], Volodymyr Porokh[b,d], Petra Kovačovicová[b,d], Martina Almáši[f], Luděk Pour[g], Martin Štork[g], Josef Havel[a,b], Sabina Ševčíková[c,f], Petr Vaňhara[b,d*]

[a]Department of Chemistry, Faculty of Science, Masaryk University, 611 37, Brno, Czech Republic

[b]International Clinical Research Center, St. Anne's University Hospital Brno, 656 91, Brno, Czech Republic

[c]Babak Myeloma Group, Department of Pathophysiology, Faculty of Medicine, Masaryk University, 625 00, Brno, Czech Republic

[d]Department of Histology and Embryology, Faculty of Medicine, Masaryk University, 625 00, Brno, Czech Republic

[e]Research Centre for Applied Molecular Oncology (RECAMO), Masaryk Memorial Cancer Institute, 602 00, Brno, Czech Republic

[f]Department of Clinical Hematology, University Hospital Brno, 625 00, Brno, Czech Republic

[g]Department of Internal Medicine, Hematology and Oncology, University Hospital Brno, 625 00, Brno, Czech Republic

*Corresponding author: Dr. Petr Vaňhara, pvanhara@med.muni.cz

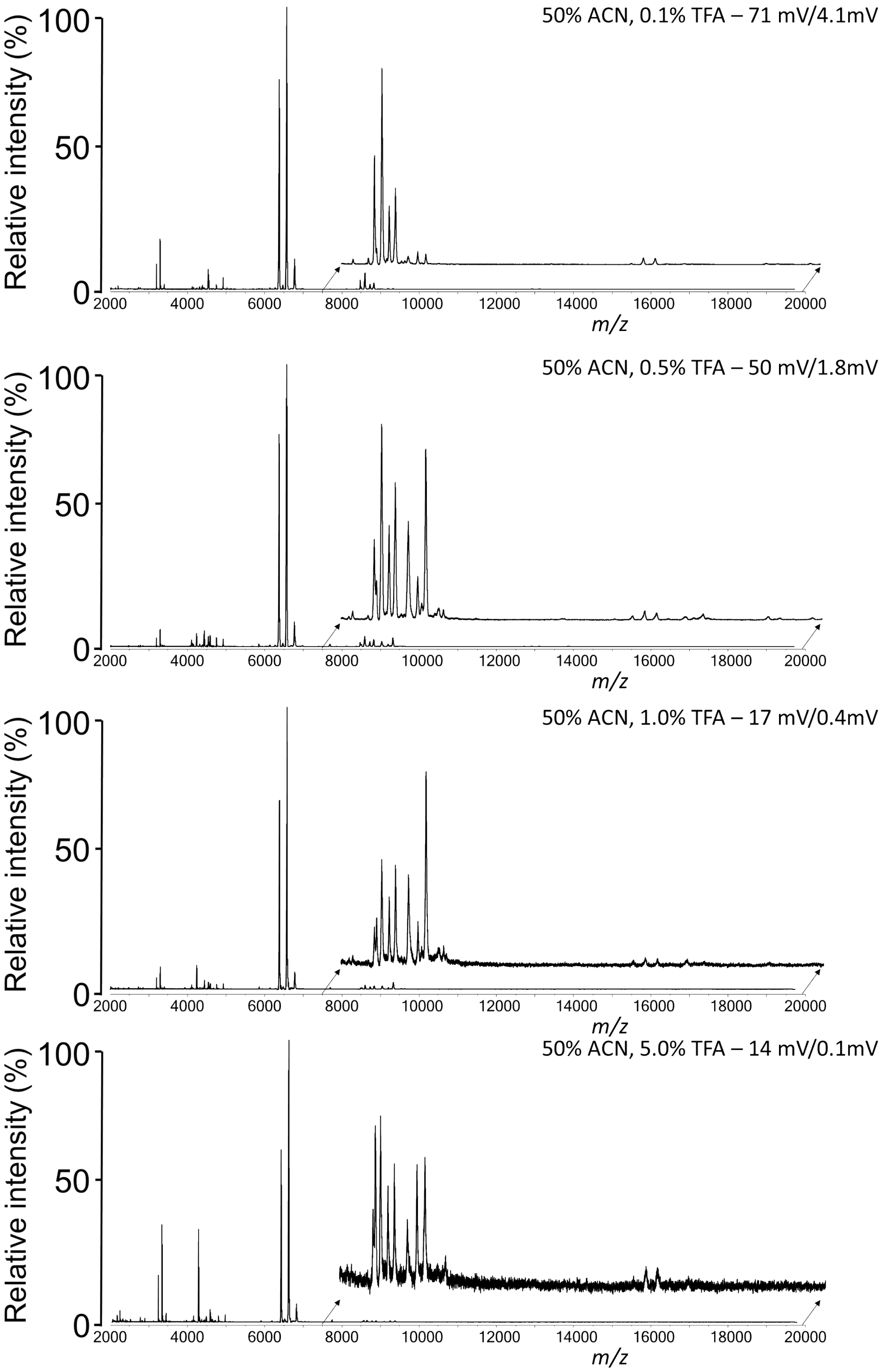

Figure S1: Effect of varying concentration of TFA in the second extraction step on quality of mass spectra of peripheral blood plasma PCL patients

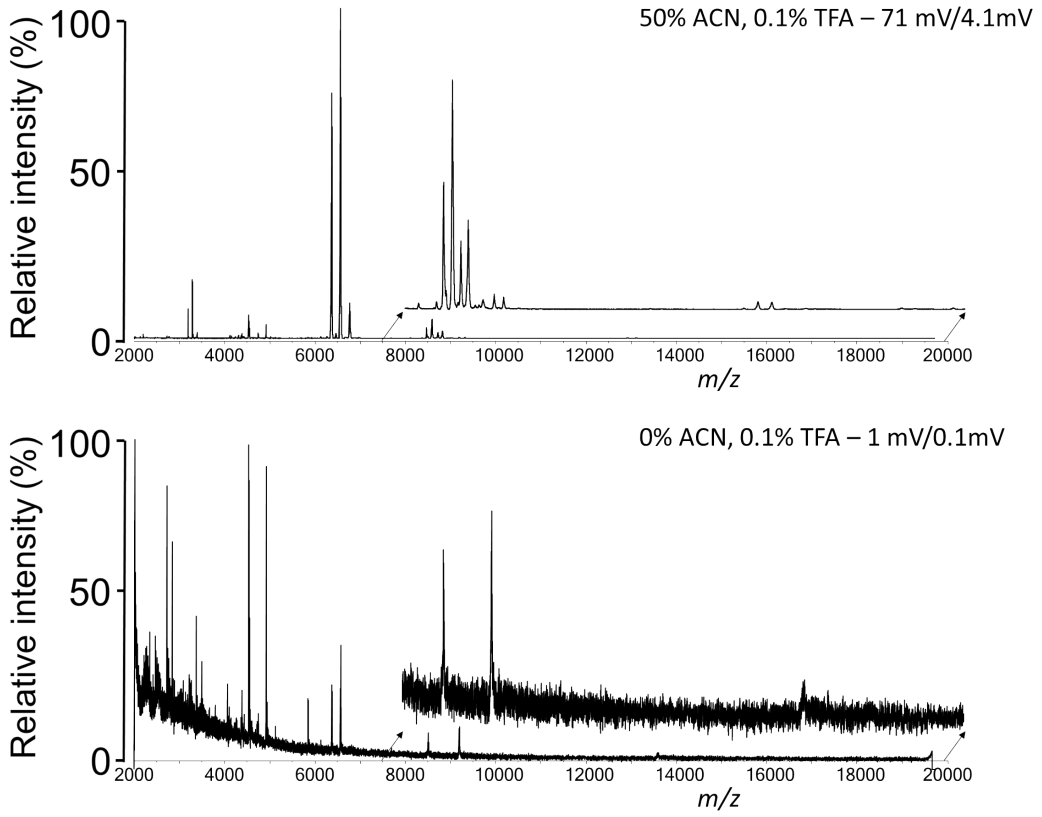

Figure S2: Effect of 50% ACN vs 0% ACN in the second extraction step on quality of mass spectra of PCL peripheral blood plasma.

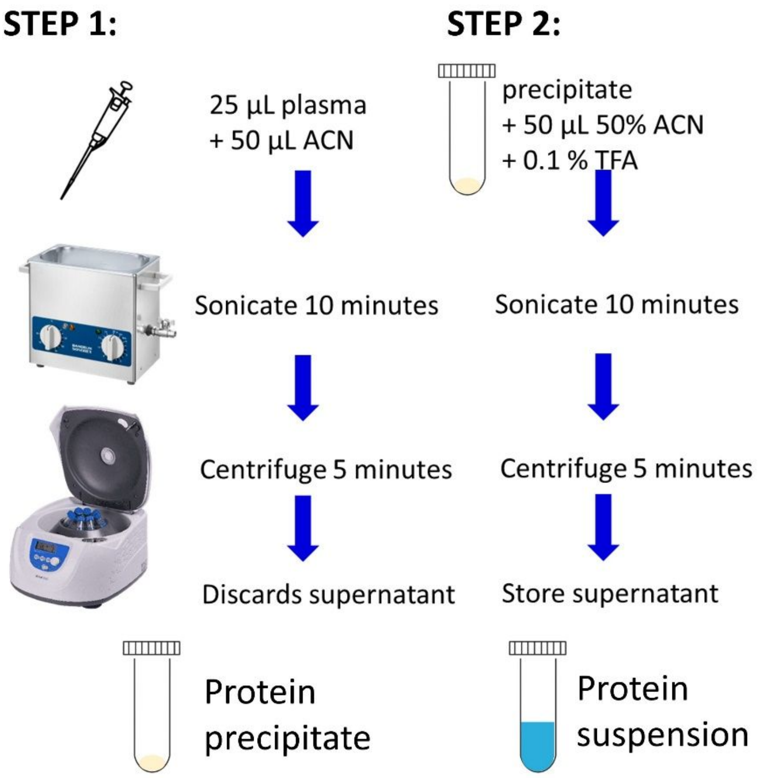

Figure S3: Graphical overview of protein extraction. An aliquot (25 µL) of peripheral blood plasma sample was added to a tube containing 50 µL of ACN, then sonicated in an ultrasonic bath for 10 min and centrifuged for 5 min at 14 500 rpm. The pellet was then mixed with 50 µL of a mixture of 50% ACN supplemented with 0.1% TFA, sonicated, and centrifuged for 5 min at 14 500 rpm. The collected supernatant (protein extract) was analyzed by MALDI TOF MS.

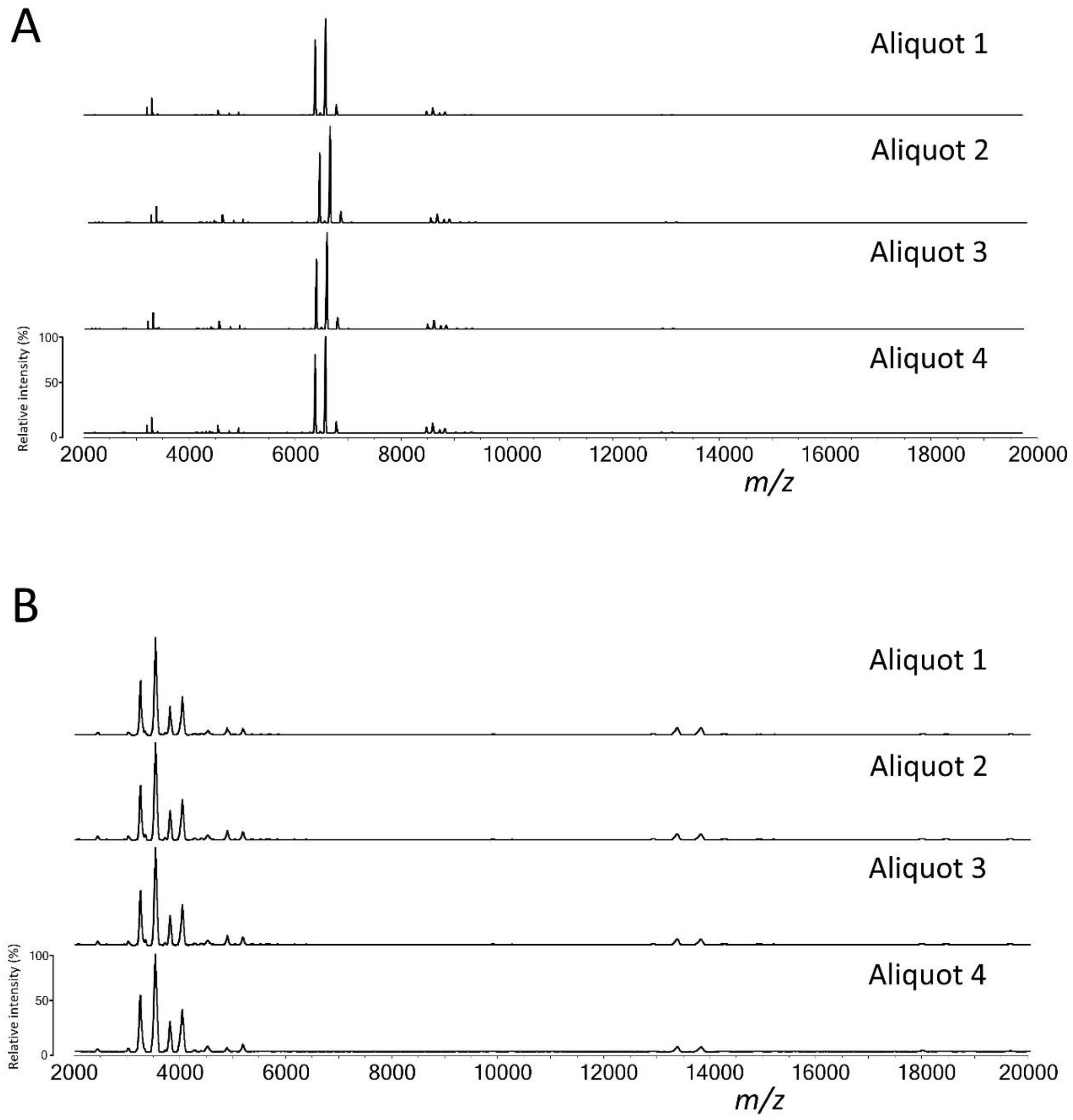

Figure S4: Documenting the reproducibility of two-step extraction protocol by visualization of mass spectra recorded from four different aliquots of peripheral blood plasma extracts from a single PCL patient. 2-20 kDa (A) and 8-16 kDa (B).
